# Acupuncture for treating chronic spinal pain: A systematic review and meta-analysis protocol

**DOI:** 10.1097/MD.0000000000032131

**Published:** 2022-12-02

**Authors:** Xingye Li, Xiao Han, Kuan Li

**Affiliations:** a Department of Spine Surgery, Beijing Jishuitan Hospital, Beijing, China; b Department of Orthopedic Surgery, Beijing Jishuitan Hospital, Beijing, China; c Department of Orthopedics, Peking Union Medical College Hospital, Chinese Academy of Medical Sciences and Peking Union Medical College, Beijing, China.

**Keywords:** acupuncture, meta-analysis, pain, protocol, spine

## Abstract

Chronic spinal pain is one of the most leading causes of disability among adults worldwide and is associated with significant health care use. The effect of acupuncture on chronic spinal pain seems controversial based on evidence-based perspective. Thus, we performed a protocol for systematic review and meta-analysis to evaluate the effectiveness of acupuncture for treatment of chronic spinal pain.

**Methods:**

This systematic review has been registered in PROSPERO (CRD42019120665), which will be conducted in accordance with preferred reporting items for systematic review and meta-analysis protocols 2015 statement. We will search 7 electronic databases to identify relevant studies from inception to November, 2022, which includes PubMed, MEDLINE, Embase, Cochrane Clinical Trials Database, Web of Science, China National Knowledge Infrastructure, and Chinese Biomedical Literature Database. Cochrane “bias risk” tool is used to assess the bias risk of the quality of the included literature. RevMan V.5.3 software will be used to analyze all data.

**Results:**

A synthesis of current evidence of acupuncture for treating chronic spinal pain will be provided in this protocol.

**Conclusion:**

This review will provide directions and recommendations for future research and clinical practices of acupuncture for treating chronic spinal pain.

## 1. Introduction

Chronic spinal pain is one of the most leading causes of disability among adults worldwide and is associated with significant health care use.^[1–3]^ It covers chronic neck, thoracic, and low back pain. Despite the frequency of this presenting complaint, a clear understanding of its etiologies is often elusive.^[4]^ Any innervated spinal structures, such as muscles, synovial joints, intervertebral discs, dura mater, and ligaments, may cause pain theoretically. Study in assessing the effect of chronic spinal pain on the US economy, found that costs were close to $86 billion. From 1997 through 2005 costs increased 65%; patients seeking spine-related care increased 49%.^[5]^

Because the diagnosis of chronic spinal pain is difficult, its treatment is usually nonspecific. Currently, nonsurgical treatments for chronic spinal pain, including nonsteroidal anti-inflammatory drugs, joint manipulation, and exercise therapy, seem to have limited benefits (small to moderate effect sizes).^[6,7]^ This finding may be explained by the fact that such treatments do not comply with recent advances in chronic pain research.

Recently, acupuncture has been a popular alternative method which is widely used in China and western countries.^[8,9]^ The effectiveness of acupuncture for treating many diseases has been reported in several clinical studies. Several high quality trials have suggest that acupuncture is effective in numerous condition. It is reported that acupuncture therapy is effective in relieving symptoms such as chronic neck pain, back pain, and lower back pain. In addition, because of the fewer effects, it is considered safe. However, some studies have reported controversial results. For example, there is no significant difference regarding pain relief in joint degeneration, fibromyalgia and rheumatoid arthritis after acupuncture therapy. ^[10–12]^ Thus, we performed a protocol for systematic review and meta-analysis to evaluate the effectiveness of acupuncture for treatment of chronic spinal pain.

## 2. Methods

### 2.1. Study registration

This systematic review has been registered in PROSPERO (CRD42019120665), which will be conducted in accordance with preferred reporting items for systematic review and meta-analysis protocols 2015 statement.^[13]^ Ethical approval is not necessary because this is a secondary research based on published data.

### 2.2. Inclusion criteria for study selection

#### 2.2.1. Types of study.

To evaluate the efficacy and safety of acupuncture for treatment of chronic spinal pain, only related randomized controlled trials (RCTs) will be included in the evaluation. Others such as case reports, animal experiments, non-RCTs, or RCT protocol will be excluded.

#### 2.2.2. Types of participants.

The patients range are limited to adult patients diagnosed with chronic spinal pain, regardless of age, gender, educational status or racial restrictions.

#### 2.2.3. Types of intervention.

Intervention groups receive acupuncture treatment and control groups receive herbal medicine, moxibustion, wet cupping or western medicine. There are no restrictions with respect to dosage, frequency, duration, or follow-up time of treatment.

#### 2.2.4. Types of outcome measures.

The primary outcomes are visual analogue scale, functional disability measured by Oswestry Disability Index. The secondary outcomes are adverse events and psychological improvements measured by scales such as the Hamilton Anxiety Rating Scale and Self-rating Anxiety Scale.

### 2.3. Search strategy

We will search 7 electronic databases to identify relevant studies from inception to November, 2022, which includes PubMed, MEDLINE, Embase, Cochrane Clinical Trials Database, Web of Science, China National Knowledge Infrastructure, and Chinese Biomedical Literature Database using the keywords “acupuncture,” “chronic pain,” and “spine.” The search strategy in PubMed is shown in Table 1. In addition, the reference lists of previously published systematic reviews were manually examined for further pertinent studies.

**Table 1:** Search strategy for the PubMed database.

#1 chronic pain [Title/Abstract]
#2 ache [Title/Abstract]
#3 discomfort [Title/Abstract]
#4 #1 OR #2 OR #3
#5 neck [Title/Abstract]
#6 cervicalgia [Title/Abstract]
#7 cervicodynia [Title/Abstract]
#8 cervical [Title/Abstract]
#9 neckache [Title/Abstract]
#10 back [Title/Abstract]
#11 backache [Title/Abstract]
#12 dorsalgia [Title/Abstract]
#13 vertebrogenic pain syndrome [Title/Abstract]
#14 #5 OR #6 OR #7 OR #8 OR #9 OR #10 OR #11 OR #12 OR #13
#15 acupuncture [Title/Abstract]
#16 electroacupuncture [Title/Abstract]
#17 fire needling [Title/Abstract]
#18 #15 OR #16 OR #17
#19 #4 AND #14 AND #18

### 2.4. Study selection

Two independent researchers screened the study titles and abstracts according to the inclusion criteria. The full text of the studies potentially meeting the eligibility criteria were retrieved for a more detailed read to make a final decision regarding inclusion.

### 2.5. Data extraction

**Figure 1.**
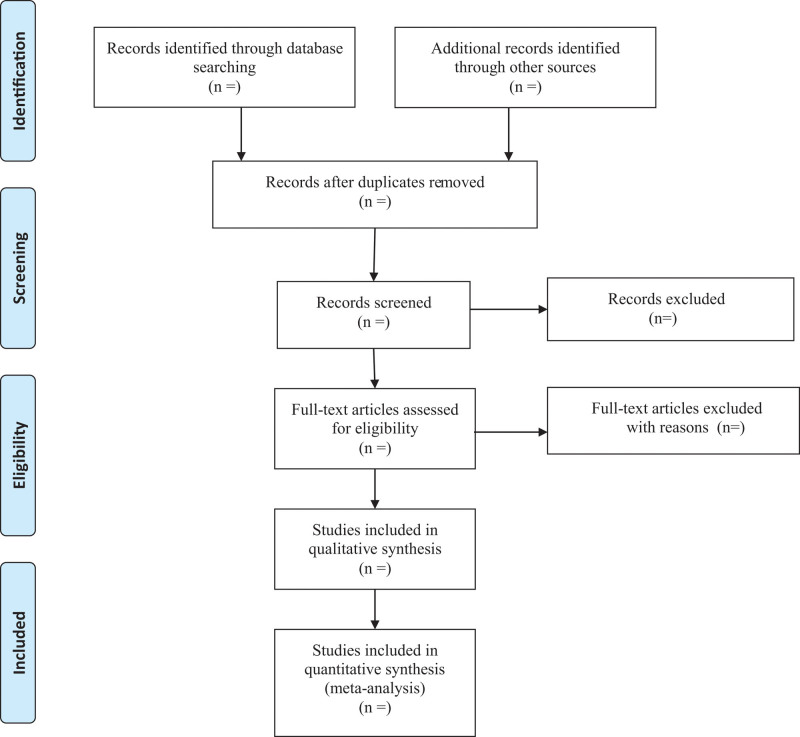
PRISMA flow chart of study selection process.

The following data are extracted: author name; publication year; country of origin; study design; sample size; age; outcome measures and complications. Any differences of opinion will be resolved through group discussion or consultation with a third reviewer. When relevant data is not reported, we will contact the author via email or other means to obtain missing data. The Preferred Report items for the System Review and Meta-analysis flow diagram (Fig. 1) will be filled out after the screening study is completed to provide specific information.

### 2.6. Risk of bias

We will use the Cochrane “bias risk” tool to assess the bias risk of the quality of the included literature.^[14]^ Assessment items included random sequence generation, allocation concealment, blinding of participants and personnel, blinding of outcome assessment, whether the incomplete outcome data were adequately handled, evidence of selective outcome reporting, and other potential sources of bias.

### 2.7. Data synthesis

RevMan V.5.3 software will be used to analyze all data. In meta-analysis, Mantel-Haenszel method will be conducted to estimate the binary outcomes effect size, while Inverse-Variance method will be conducted to estimate the continuous outcomes effect size. We will use the fixed-effect model to pool data whenever there is low heterogeneity. Analysis and treatment will be carried out first whenever there is high heterogeneity (*P* < .1 or *I*^2^ > 50%). If it cannot be solved, the random-effect model will be introduced to provide a more conservative effect estimation. For the research results with large heterogeneity that cannot be quantitatively integrated, a narrative report will be made. Sources of heterogeneity were assessed by sensitivity analysis, after excluding studies of low quality or small sample size, if the heterogeneity did not change significantly, the results were robust. Otherwise, the excluded studies may have been source of heterogeneity. In this study, fewer than 10 included studies were evaluated for publication bias using funnel plot, otherwise Egger regression test would be used.

### 2.8. Assessment of quality of evidence

We will use the Grading of Recommendations Assessment, Development, and Evaluation to assess the results.^[15]^ In the Grading of Recommendations Assessment, Development, and Evaluation system, the quality of evidence will be categorized into 4 levels: high, moderate, low, and very low quality.

## 3. Discussion

Three of the most common musculoskeletal conditions associated with the spine (cervical, thoracic, and lumbar) are chronic uncomplicated neck pain, back pain, and lower back pain, with respective incidences of 18%, 17.7%, and 36%.^[16–18]^ These 3 conditions negatively influence patient quality of life, producing substantial economic and social burdens.

As one part of traditional Chinese medicine, acupuncture therapy has been widely used in clinical trials of pain. On the basis of traditional Chinese medicine theory, acupuncture can regulate the balance of *qi* and blood by stimulating acupuncture points, which aims at improving physiological function. Meanwhile, vast studies have shown that acupuncture tianshu (ST25), zusanli (ST36), and taichong (LR3) can adjust the production of neurotransmitter, mainly 5-hydroxytryptamine, and reduce nerve sensitivity.^[19]^ As an analgesic form of therapy, the effect of acupuncture has been considered as an overall safe alternative therapy in various studies, with few side effects (harmless) and without any environmental impact.^[20]^ Therefore, it could also be considered as a “sustainable” treatment form. This study provides a reference basis for acupuncture in treating chronic spinal pain.

## Author contributions

**Conceptualization:** Kuan Li.

**Data curation:** Kuan Li.

**Methodology:** Xiao Han.

**Writing – original draft:** Xingye Li.
